# GluOC Induced SLC7A11 and SLC38A1 to Activate Redox Processes and Resist Ferroptosis in TNBC

**DOI:** 10.3390/cancers17050739

**Published:** 2025-02-21

**Authors:** Jiaojiao Xu, Xue Bai, Keting Dong, Qian Du, Ping Ma, Ziqian Zhang, Jianhong Yang

**Affiliations:** Medical School, University of Chinese Academy of Sciences, No. 1, Yanqi Lake East Road, Huairou District, Beijing 101408, China; xujiaojiao18@mails.ucas.ac.cn (J.X.); baixue22@mails.ucas.ac.cn (X.B.); dongketing21@mails.ucas.ac.cn (K.D.); duqian22@mails.ucas.ac.cn (Q.D.); maping23@mails.ucas.ac.cn (P.M.); zhangziqian23@mails.ucas.ac.cn (Z.Z.)

**Keywords:** TNBC, osteocalcin, SLC7A11, SLC38A1, ferroptosis

## Abstract

Background/Objectives: Ferroptosis, a type of programmed cell death, is mainly associated with disruptions in iron metabolism, imbalances in the amino acid antioxidant system, and the build-up of lipid peroxides. Triple-negative breast cancer (TNBC) has a dismal prognosis. Since activating ferroptosis can suppress breast cancer cell proliferation, it holds promise as a novel therapeutic target for breast cancer patients. Thus, the objective of this study was to clarify the mechanism of ferroptosis in TNBC, aiming to find new treatment strategies for TNBC patients. Methods: We screened out the differential genes related to ferroptosis in TNBC after GluOC treatment based on the whole-genome sequencing results. At the cellular level, we conducted explorations using techniques such as quantitative real-time polymerase chain reaction (qRT-PCR), Western blotting, fluorescence staining, and siRNA transfection. Moreover, we further verified the role of GluOC in inhibiting ferroptosis in TNBC through in vivo experiments using nude mice. Results: The results showed that GluOC enhanced glutathione expression levels by inducing SLC7A11 accumulation via the specific signaling pathway. Additionally, GluOC increased ATP production and tricarboxylic acid flux resistance to ferroptosis through SLC38A1. Overall, GluOC coordinately regulated SLC7A11 and SLC38A1 to inhibit ferroptosis in TNBC. Conclusions: This study elucidated the mechanism of GluOC in inhibiting ferroptosis in TNBC. The findings not only provided new insights into ferroptosis but also potentially offered new concepts for the development of future anticancer therapies, which may contribute to improving the treatment of TNBC patients.

## Simple Summary

Triple-negative breast cancer (TNBC) has a poor prognosis. This study was proposed to explore new treatment approaches for TNBC. The author aimed to understand the role of ferroptosis in TNBC. The study found that uncarboxylated osteocalcin (GluOC) can inhibit ferroptosis in TNBC by regulating SLC7A11 and SLC38A1. This discovery may provide new ideas for developing anticancer therapies and contribute to the research on TNBC treatment in the field.

## 1. Introduction

Breast cancer (BC) is a growing global healthcare issue. The World Health Organization reported that in 2022, there were ~20 million new cases of cancer worldwide, with BC as the second most common type of cancer. BC has a high incidence among women and is a leading cause of death [1]. The incidence of BC in China is increasing by 3–4% per year [2]. Among the different types of BC, due to the lack of estrogen receptor (ER), progesterone receptor (PR), and human epidermal growth factor receptor 2 (HER2), triple-negative breast cancer (TNBC) has the highest mortality rate, a higher recurrence rate, more frequent metastasis, and a lack of sensitivity to current hormone therapies and other treatment methods; therefore, the search for novel treatment targets is necessary to develop new therapeutic strategies for TNBC [3].

Ferroptosis is a mode of programmed cell death first proposed by Dixon et al. [4]. Inducing ferroptosis offers potential promise for cancer treatment, particularly for invasive or treatment-resistant cancers such as TNBC [5]. In TNBC cells, the main features of ferroptosis include antioxidant defense mechanisms. xCT/solute carrier family 7 member 11 (SLC7A11) overexpression promotes the production of glutathione (GSH) and the proliferation of TNBC cells [6]. The availability of amino acids also helps certain types of tumors, including TNBC, resist susceptibility to ferroptosis [7]. The solute carrier family 38 member 1 (SLC38A1) glutamine transporter is a member of a transporter superfamily that provides metabolic fuel or acts as precursor for glutathione synthesis and serves a carcinogenic role in the development of BC [8].

Osteocalcin (OC) is a non-collagen protein secreted by osteoblasts. Uncarboxylated osteocalcin (GluOC) is an endocrine hormone that regulates glucose and lipid metabolism and promotes testosterone synthesis and cognitive function [9]. It has been previously reported that serum osteocalcin in breast cancer patients with bone metastasis is higher than that in healthy people. OC promotes proliferation and migration of prostate cancer through GPRC6A and has the potential to increase pancreatic cancer cell growth and invasion. In addition, experimentally-induced reduction in the number of Ocn+ cells suppresses both the neutrophil response and lung tumor outgrowth [10,11,12,13,14]. Our previous study reported that GluOC enhanced the expression of TNBC bone-lytic transfer genes [15], as well as promoting the proliferation and inhibiting the apoptosis of TNBC cells [16].

The present study aimed to investigate the tumor-promoting mechanism of GluOC, focusing on its ferroptosis resistance process. It was demonstrated that GluOC inhibited iron death in TNBC and promoted the growth and metastasis of TNBC cells. GluOC increased the expression levels of SLC7A11 and glutathione peroxidase 4 (GPX4) through the WW domain-containing E3 ubiquitin protein ligase 1 (WWP1)/PTEN/phosphatidylinositol-4,5-bisphosphate 3-kinase catalytic subunit α (PIK3CA)/AKT signaling pathway, activated GSH expression in TNBC, and inhibited ferroptosis. Additionally, GluOC increased the expression levels of SLC38A1, which promoted the production of GSH and increased the α-ketoglutaric acid (α-KG) content; this increased the tricarboxylic acid (TCA) flux. Our results have great significance for further understanding the mechanism of TNBC anti- ferroptosis.

## 2. Materials and Methods

### 2.1. Transcriptome Sequencing and Data Processing

BC clinical data were obtained from the Kaplan–Meier plotter (https://kmplot.com/analysis/, 11 December 2024), and RNA sequencing data in Supplementary Materials (supplementary method) were obtained from Shanghai Applied Protein Technology Co., Ltd (Shanghai, China). Total RNA was extracted from MDA-MB-231 breast cancer cell using TRIzol^®^. Paired-end libraries were prepared using an ABclonal mRNA-seq Lib Prep Kit (RK20350, ABclonal, Wuhan, China) following the manufacturer’s instructions. Sequencing was performed with an Illumina Novasea 6000/MGISEO-T7 instrument (Illumina, CA, USA). The data generated from Illumina/BGl platform were used for bioinformatics analysis.

### 2.2. Cell Culture

The SKBR3, MCF7, MDA-MB-231, and MDA-MB-468 BC cell lines were obtained from The Cell Bank of Type Culture Collection of The Chinese Academy of Sciences and were maintained in DMEM (Gibco; Thermo Fisher Scientific, Inc., Waltham, MA, USA) containing 10% FBS (Viva Cell BIOSCIENCES, XP BioMed, Shanghai, China) in 5% CO_2_ at 37 °C.

### 2.3. Transfections

The siRNAs were purchased from Sangon Biotech Co., Ltd. (Shanghai, China). A 6-well plate was seeded with 1 × 10^5^ cells, and siRNA transfection was performed 24 h later using Lipofectamine™ 2000 reagent (Yeasen Biotechnology Co., Ltd., Shanghai, China). The cells were collected after 24 h of culture in a medium containing 10% FBS without double antibiotics (penicillin and streptomycin). The siRNAs are shown in Table 1.

### 2.4. Propodium Iodide (PI) Staining

The cells were incubated in a 6-well plate, and ~80% of the cell volume was transfected with siRNAs. After 24 h, the DMEM containing 10% FBS without double antibiotics was replaced, and GluOC (160 ng/mL) was added. After 24 h, the cells were collected. After 3 rounds of washing with PBS, 1× PI staining buffer was added (cat. no. CA1020; Solarbio Science & Technology Co., Ltd., Beijing, China), and after 5 min of incubation in the dark, anti-quenching fluorescent tablets containing DAPI were added, and samples were imaged using a fluorescence microscope.

### 2.5. Wound-Healing Assay

The cells were cultured in complete medium to ~90% confluency. Subsequently, the cells were scraped with the tip of a 10 μL pipette and washed 3 times to remove cell debris; then, images of the wound at 0 h were recorded. The gene knockout was performed, and cells were incubated for 24 h. Subsequently, GluOC (160 ng/mL) was added to the cells, and samples were imaged after 24 h (ZOE Fluorescent Cell Imager, Bio-Rad Laboratories, Inc., Hercules, CA, USA). The migration rate was calculated as (D0 − Dt)/D0, where D0 was the initial distance between the wound at 0 h, and Dt was the distance between the wound at 48 h.

### 2.6. Migration Assay

After transfection, 3 × 10^4^ cells were added to the Transwell chamber and cultured in basic medium. Medium containing 10% FBS and GluOC (160 ng/mL) was added to the lower chamber. The cells were immobilized, stained with crystal violet, viewed using a microscope, and counted using ImageJ 1.52a software (National Institutes of Health, Bethesda, MD, USA).

### 2.7. Colony Formation Assay

Following transfection, 100 cells/well were seeded in 12-well plates and cultured at 37 °C for 14 days. The colonies were fixed with 4% paraformaldehyde and stained with crystal violet.

### 2.8. Lipid Reactive Oxygen Species (ROS) Assay

Cells were stained with BODIPY 581/591 C11 (cat. no. GC40165; GlpBio, Shanghai, China). GluOC (160 ng/mL) was added, and cells were washed 3 times with PBS and incubated at 37 °C for 30 min in medium containing 2 μM BODIPY 581/591 C11. Cells were washed twice with PBS and imaged using a fluorescence microscope.

### 2.9. Iron Assay

Transfected cells were treated with GluOC (160 ng/mL) and incubated for 24 h. The cells were then washed with a base medium. FerroOrange (cat. no. F374; Dojindo Laboratories, Inc., Kumamoto, Japan) at a concentration of 1 µmol/l was added, and cells were incubated in 5% CO_2_ at 37 °C for 15 min. Cells were imaged using a fluorescence microscope.

### 2.10. ROS Assay

Intracellular ROS levels were measured using 10 μM diacetyldichlorofluorescein (DCFH-DA; cat. no. 4091-99-0; Macklin, Shanghai, China). TNBC cells were incubated with the fluorescent probe at 37 °C for 30 min and imaged using fluorescence microscopy and flow cytometry. The final result was calculated as a percentage of the control.

### 2.11. Malondialdehyde (MDA) Assay

TNBC cells were seeded in culture dishes for 24 h. Cells were transfected and then treated with GluOC (160 ng/mL). The cells were collected, the absorbance of the samples at 532 nm was measured using a kit (cat. no. BC0025; Solarbio Science & Technology Co., Ltd., Beijing, China), and the MDA content in the cells was measured.

### 2.12. GSH Assay

TNBC cells were incubated in 6-well plates, transfected, and treated with GluOC (160 ng/mL). Cells were collected, and the GSH/glutathione sulfate (GSSH) content was measured using a kit (cat. no. E-BC-K097-M; Elabscience Biotechnology, Inc., Wuhan, China). The absorbance of the samples at 412 nm was measured by enzyme labelling.

### 2.13. Flow Cytometry

MDA-MB-231 BC cells were seeded in 6-well plates (2 × 10^5^ cells/well). The cells were transfected, treated with GluOC, and incubated for 24 h. Cells were harvested using trypsin and incubated at 37 °C for 30 min in the dark with DCFH-DA. The cellular oxidative stress levels were then analyzed by flow cytometry (FACSCalibur; BD Biosciences, Milpitas, CA, USA).

### 2.14. Measurement of Mitochondrial Membrane Potential (MMP)

The MMP assay kit (cat. no. J22202; LABLEAD Trading CO., LTD, Beijing, China) was used to evaluate the changes in MMP. The working solution was prepared by adding 1 mL of dye buffer to 5 µL JC-1 (200×). The solution was thoroughly mixed with the cells, and samples were incubated at 37 °C for 30 min. After washing, 2 mL of whole medium was added, and the samples were imaged using a fluorescence microscope.

### 2.15. α-KG Content Detection

Cells were seeded at 5 × 10^5^ in a 6-well plate, transfected with siRNA for 24 h, and then treated with GluOC (160 ng/mL) for 24 h. Cells were washed three times with PBS, and, after being cleaned three times with PBS, α-KG content detection kit (cat. no. BC5425; Solarbio Science & Technology Co., Ltd., Beijing, China) was used. The absorbance at 340 nm was detected.

### 2.16. ATP Content Detection

Intracellular ATP content was detected using the ATP content detection kit (cat. no. BC0305; Solarbio Science & Technology Co., Ltd., Beijing, China). The absorbance at 340 nm was detected.

### 2.17. Detection of Glutamine Content

Cells were transfected and treated with GluOC (160 ng/mL). Then, cells were harvested, and 0.2 mL saline (0.9% NaCl) was added for mechanical homogenization. Cells were then centrifuged at 10,000× *g* at 4 °C for 15 min; the supernatant was collected and centrifuged at 14,000× *g* at 4 °C through a 50 KD ultrafiltration centrifuge tube for 10 min, and then the filtrate was collected for analysis using a kit (cat. no. E-BC-K853-M; Elabscience Biotechnology, Inc., Wuhan, China).

### 2.18. Protein Stability and Ubiquitination Assays

Cells were treated with GluOC (160 ng/mL) and a 10 μM protease inhibitor MG-132 (cat. no. HY-13259; MCE, Shanghai, China) for 8 h. Then, protein was collected. Subsequently, the extracted protein serum was incubated with PTEN (1:200, cat. no. 9559T; Cell Signaling Technology, Inc., Danvers, MA, USA) and IgG antibodies (1:200, cat. no. ab133470; Abcam, MA, USA) at 4 °C for 8 h. Protein A/G + agarose beads (cat. no. P001-2, Shanghai 7sea bictech CO., LTD, Shanghai, China) were added and incubated at 4 °C overnight. The antibody PTEN (1:1000) was then used overnight at 4 degrees. The level of PTEN protein ubiquitin (1:1000, cat. no. sc-8017; Santa Cruz Biotechnology, Inc., Paso Robles, CA, USA) was determined by Western blotting the following day.

### 2.19. Western Blotting

Proteins were extracted from the cells by lysis. Subsequently, the proteins were subjected to SDS-PAGE, and the isolated protein was transferred to a polyvinylidene fluoride membrane (MilliporeSigma, Burlington, MA, USA). Cells were incubated with skim milk (5%) at room temperature for 2 h, followed by incubation with primary antibodies at 4 °C overnight. Membranes were then washed with TBST 3 times and incubated with IgG (1:500, cat. no. S0101; Beijing LABLEAD Trading CO., LTD, Beijing, China) secondary antibodies at room temperature for 1 h. β-actin (1:1000, cat. no. 81115-1-RR, Proteintech Group, Inc., Wuhan, China) was used as the internal control. Protein bands were visualized using an ECL kit (cat. no. E1070; Beijing LABLEAD Trading CO., LTD, Beijing, China). The antibodies used were as follows: SLC7A11 (1:500, cat. no. TD12509; Abmart Pharmaceutical Technology Co., Ltd., Shanghai, China), GPX4 (1:500, cat. no. 67763-1-Ig; Proteintech Group, Inc., Wuhan, China), nuclear factor erythroid 2-related factor 2 (Nrf2; 1:500, cat. no. TA0639S; Abmart Pharmaceutical Technology Co., Ltd., Shanghai, China), WWP1 (1:500, cat. no. 67804-1-Ig; Proteintech Group Inc., Wuhan, China), PTEN (1:1000, cat. no. 9559T, Cell Signaling Technology, Inc., MA, USA), PIK3CA (1:500, cat. no. bs-2067R; BIOSS), phosphorylated (P)-PIK3CA (1:500, cat. no. bs-5570; BIOSS, Beijing, China), AKT (1:500, cat. no. T55561; Abmart Pharmaceutical Technology Co., Ltd., Shanghai, China), P-AKT (1:1000, cat no. 4060; Cell Signaling Technology, Inc., MA, USA), and SLC38A1 (1:500, cat. no. 28632-1-AP; Proteintech Group, Inc., Wuhan, China).

### 2.20. Reverse Transcription-Quantitative (q)PCR

Cells were collected, and 1 mL Trizol reagent (cat no. R401-01, Nanjing Vazyme Biotechnology Co., LTD., Nanjing, China) was added. The cells were centrifuged at 12,000 rpm for 15 min at 4 °C, and 400 μL of the upper water phase was added to isopropyl alcohol, mixed, and centrifuged at 12,000 rpm for 10 min at 4 °C. The supernatant was removed, and the RNA was washed with 1 mL of 75% ethanol and precipitated twice. The purity and quality of RNA were examined using a NanoDrop2000 (Thermo Fisher Scientific, Inc.). A total of 1 µg of RNA was reverse transcribed into cDNA using the Hifaire lll lst Strand cDNA Synthesis SuperMix for qPCR (gDNA digester plus; cat. no. 11141ES60). The SYBR Green qPCR kit was used (cat. no. AQ132-24; TransGen Biotech. Co., Ltd., Beijing, China). Gene expression levels were analyzed using the 2^−ΔΔCt^ quantitative method. β-actin was used as the internal control, and the primer sequences are shown in Table 2.

### 2.21. Mouse Xenograft Model

Nude mice (BALB/C, 6-week-old female) were purchased from Beijing Lank Biotechnology Co., Ltd. (Experimental Animal Production Licence No.: SCXK (Beijing, China) 2019-0010). Animal studies were carried out according to the Guide for the Care and Use of Laboratory Animals (eighth edition, 2011) and with the approval of the Animal Care and Use Committee of the University of Chinese Academy of Sciences. During modeling, 5 × 10^5^ MDA-MB-231 cells were suspended in a mixture of PBS and Matrigel (1:1 *v*/*v*) and subcutaneously injected into the right flank of nude mice. The tumor size was recorded with vernier calipers. When the maximum tumor volume was 2000 mm^3^, the mice were sacrificed by cervical dislocation for further experiments.

### 2.22. Hematoxylin and Eosin (H&E) Staining and Immunohistochemistry

Paraffin-embedded tissue chip sections (10 μm) were prepared for H&E staining and immunohistochemical detection. The expression levels of target proteins were observed using a microscope (DM6B; Leica, Wetzlar, Germany).

### 2.23. Statistical Analysis

Statistical analysis was performed using GraphPad Prism statistical software (version 10; Dotmatics, San Diego, CA, USA). The differences among multiple groups were compared by one-way ANOVA followed by Tukey’s post hoc test, while those between two groups were compared by unpaired Student’s *t*-tests. Data were acquired from three independent assays and expressed as mean ± standard deviation. *p* < 0.05 was considered to indicate a statistically significant difference.

## 3. Results

### 3.1. GluOC Promoted the Expression of SLC7A11 and SLC38A1 Genes in Breast Cancer Cells

**Figure 1:**
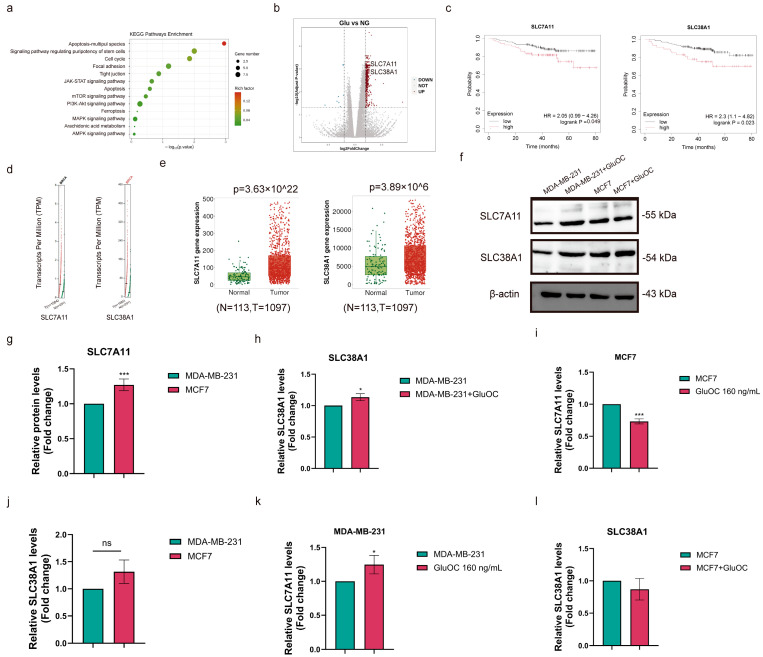
GluOC inhibited the ferroptosis process in MDA-MB-231 BC cells. (**a**) KEGG in MDA-MB-231 BC cells. Red squares indicate the ferroptosis process. (**b**) DEGs in MDA-MB-231 BC cells. (**c**) Clinical outcomes were markedly increased in patients with TNBC with high SLC7A11 and SLC38A1 expression levels according to the Kaplan–Meier and GEPIA database. (**d**) The expression levels of SLC7A11 and SLC38A1 in BC according to the GEPIA scatter diagram. (**e**) The expression levels of SLC7A11 and SLC38A1 in BC according to the Kaplan–Meier box plot. (**f**–**l**) The protein expression levels of SLC7A11 and SLC38A1 were measured by Western blotting after treatment with GluOC. Data are presented as mean ± SD (n = 3). * *p* < 0.05, *** *p* < 0.001. BC, breast cancer; GluOC, uncarboxylated osteocalcin; DEG, differentially expressed gene; TNBC, triple-negative BC; GEPIA, gene expression profiling interactive analysis; KEGG, Kyoto Encyclopedia of Genes and Genomes; SLC38A1, solute carrier family 38 member 1; SLC7A11, solute carrier family 7 member 11.

Based on the significant enrichment of DEGs in the KEGG pathway (*p* ≤ 0.05; Figure 1a), SLC7A11 and SLC38A1 expression levels were significantly elevated in the GluOC-treated group (Figure 1b). Kaplan–Meier data show that among patients with BC, the high-SLC7A11-expression and high-SLC38A1-expression groups of patients demonstrated worse OS prognostic indicators (Figure 1c). Moreover, the expression levels of SLC7A11 and SLC38A1 in BC tissues were higher compared with those in normal tissues (Figure 1d,e).

Western blotting results showed that SLC7A11 protein expression levels were highest in MCF7 cells compared with MDA-MB-231 cells (Figure 1f, The uncropped blots are shown in Figure S1A). GluOC treatment significantly increased the expression levels of SLC7A11 in MDA-MB-231 cells and decreased the expression levels of SLC7A11 in MCF7 cells (Figure 1g,i). SLC38A1 expression showed no significant difference between MDA-MB-231 and MCF7 cells (Figure 1j). After GluOC treatment, SLC38A1 expression levels in MDA-MB-231 cells were significantly increased, but there was no significant effect on MCF7 cells (Figure 1k–l). Given that GluOC significantly increased SLC7A11 and SLC38A1 levels in MDA-MB-231 cells, TNBC was selected as the focus for the present study.

### 3.2. GluOC Inhibits Ferroptosis in TNBC Cells

System Xc- (including SLC7A11 and SLC3A2)-mediated cystine absorption plays an important role in ferroptosis. Inhibiting system XC- obstructs the absorption of cystine, while glutathione is a necessary cofactor for the function of GPXs, resulting in a decrease in GPXs’ activity, a decline in cell anti-peroxidation ability, and an accumulation of lipid reactive oxygen species, resulting in cell oxidative death [17].

**Figure 2:**
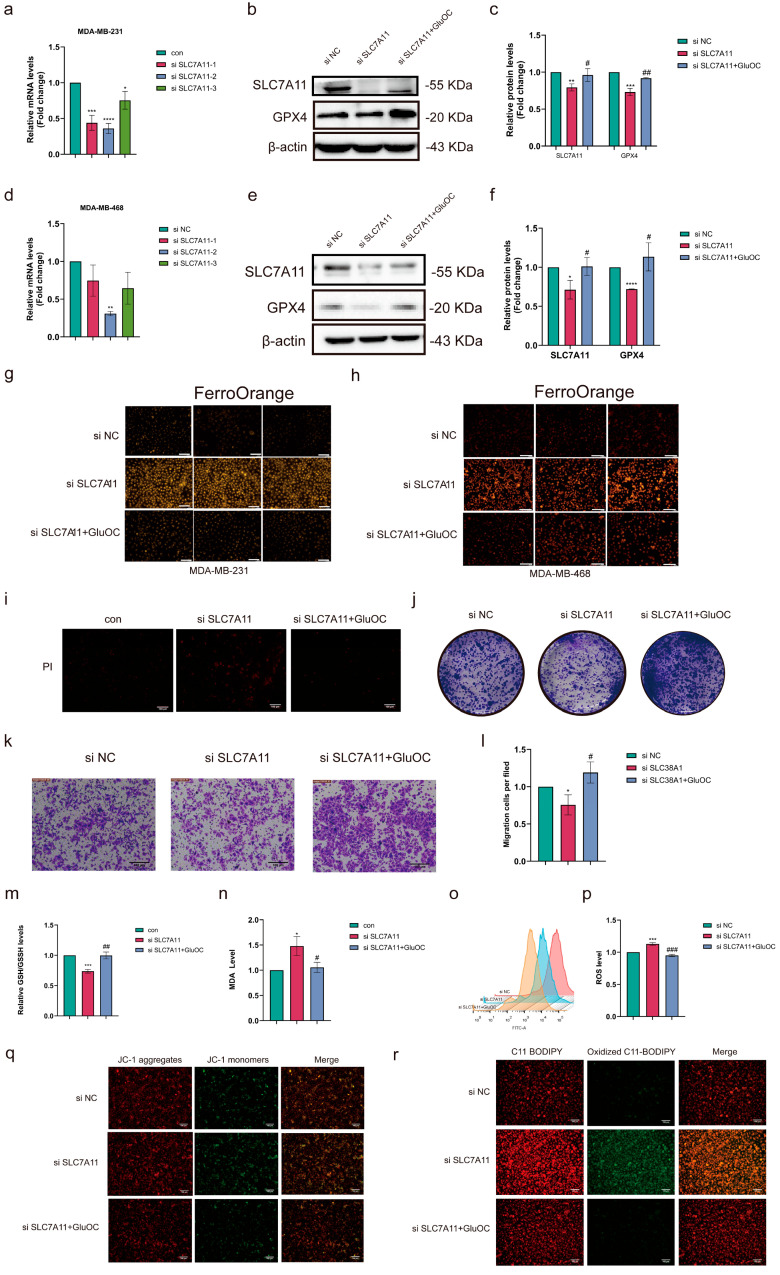
GluOC increased the GSH content in TNBC by increasing SLC7A11. (**a**) Expression levels of SLC7A11 in MDA-MB-231 cells were measured by RT-qPCR after treatment with si SLC7A11. (**b**) The protein expression levels of SLC7A11 in MDA-MB-231 cells measured by Western blotting after treatment with si SLC7A11 and/or GluOC. (**c**) Quantification of Western blotting results. (**d**) Expression level of SLC7A11 in MDA-MB-231 cells measured by RT-qPCR after treatment with si SLC7A11. (**e**) Protein level of SLC7A11 in MDA-MB-468 cells measured by Western blotting after treatment with si SLC7A11 and/or GluOC. (**f**) Quantification of Western blotting results. (**g**) After a 24 h pretreatment with si SLC7A11, MDA-MB-231 cells were irradiated with or without GluOC for 24 h, and the intracellular Fe2+ concentration was measured (scale bar = 100 μm). (**h**) After a 24 h pretreatment with si SLC7A11, MDA-MB-468 cells were irradiated with or without GluOC for 24 h, and the intracellular Fe2+ concentration was measured (scale bar = 100 μm). (**i**) GluOC alleviated the death of MDA-MB-231 cells induced by SLC7A11 knockdown (scale bar = 100 μm). (**j**) The clonogenicity of stably transfected MDA-MB-231 cells indicated cell proliferation. (**k**) Transwell assays of MDA-MB-231 cells treated with si SLC7A11 and GluOC for 24 h (scale bar = 100 μm). (**l**) Quantitative analysis of Transwell. (**m**) Quantification of the GSH/GSSH levels in MDA-MB-231 cells. (**n**) Quantification of the MDA levels in MDA-MB-231 cells. (**o**) The ROS level was detected by flow cytometry. (**p**) Quantitative analysis of ROS. (**q**) GluOC alleviated the MMP of MDA-MB-231 cells induced by SLC7A11 knockdown (scale bar = 100 μm). (**r**) Lipid peroxidation was measured using C11 BODIPY 581/591 in MDA-MB-231 cells after treatment with SLC7A11 knockdown and GluOC (scale bar = 100 μm). Data are presented as the mean ± SD (n = 3). * *p* < 0.05, ** *p* < 0.01, *** *p* < 0.001, **** *p* < 0.0001. con vs. si SLC7A11. # *p* <0.05, ## *p* <0.01, ### *p* <0.001 si SLC7A11 vs. GluOC. Si, small interfering RNA; SLC7A11, solute carrier family 7 member 11; RT-qPCR, reverse transcription-quantitative PCR; GluOC, uncarboxylated osteocalcin; con, control.

In order to investigate the role of GluOC in ferroptosis, the expression of SLC7A11 was knocked down. These results showed that MDA-MB-231 and MDA-MB-468 cells were more sensitive to si SLC7A11, and the knockdown efficiency was the highest (Figure 2a,d). GluOC mitigated the effect of gene knockdown and increased the expression levels of SLC7A11 and GPX4 in MDA-MB-231 (Figure 2b,c. The uncropped blots are shown in Figure S1B) and MDA-MB-468 cells (Figure 2e,f. The uncropped blots are shown in Figure S1C). FerroOrange fluorescent probes were used to measure intracellular Fe^2+^ content in MDA-MB-231 and MDA-MB-468 cells, which showed that the knockout of SLC7A11 significantly increased intracellular Fe^2+^ levels and that the addition of GluOC effectively inhibited the effect of iron accumulation induced by gene knockdown (Figure 2g,h). Subsequently, the mechanism of ferroptosis was explored in MDA-MB-231 cells. PI fluorescence staining showed that SLC7A11 deletion caused cell death, which was reversed by GluOC treatment (Figure 2i). Decreasing the expression levels of SLC7A11 reduced the proliferation of MDA-MB-231 cells, and conversely, the addition of GluOC interfered with the inhibition of si SLC7A11 on cancer cells (Figure 2j). Transwell assay results showed an increased number of migrating cells in the GluOC-treated group compared with the knockdown group (Figure 2k,l). In addition, the decrease in SLC7A11 expression resulted in the decrease in intracellular GSH/GSSH content, while the addition of GluOC increased GSH content (Figure 2m). GluOC treatment reversed the increase in MDA levels caused by si SLC7A11 (Figure 2n). Additionally, si SLC7A11 significantly increased cellular ROS levels, and GluOC treatment reduced the cellular damage caused by si SLC7A11 (Figure 2o,p). MMP (Figure 2q) and lipid ROS (Figure 2r) are the core products of ferroptosis. The levels of MMP and ROS in the si SLC7A11 group were significantly higher compared with those in the control group, while GluOC treatment mitigated the effects of si SLC7A11. These results suggested that GluOC increased SLC7A11 and GPX4 expression in MDA-MB-231 BC cells, thereby increasing GSH levels and inhibiting ferroptosis in TNBC cells.

### 3.3. GluOC Regulates SLC7A11-Induced Ferroptosis of MDA-MB-231 Breast Cancer Cells via Nrf2

**Figure 3:**
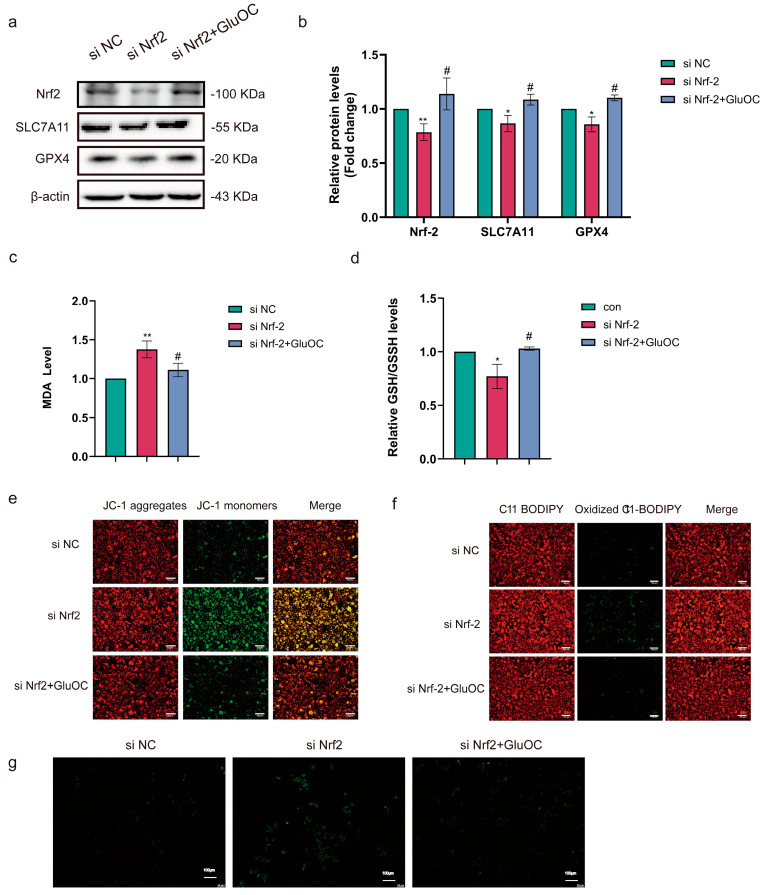
GluOC increases the expression levels of Nrf2/SLC7A11/GPX4 in MDA-MB-231 breast cancer cells. (**a**) GluOC decreased the expression levels of Nrf2, SLC7A11, and GPX4 in MDA-MB-231 cells induced by Nrf2 knockdown. (**b**) Western blotting used ImageJ software for density quantification. (**c**) Quantification of the MDA levels in MDA-MB-231 cells. (**d**) Quantification of the GSH/GSSH levels in MDA-MB-231 cells. (**e**) GluOC decreased the MMP levels of MDA-MB-231 cells induced by Nrf2 knockdown (scale bar = 100 μm). (**f**) Lipid peroxidation was measured using C11 BODIPY 581/591 in MDA-MB-231 cells after treatment with Nrf2 knockdown and GluOC (scale bar = 100 μm). (**g**) DCFH-DA staining (scale bar = 100 μm). Data are presented as the mean ± SD (n = 3). * *p* < 0.05, ** *p* < 0.01, con vs. si Nrf2. # *p* < 0.05, si Nrf2 vs. GluOC. MDA, malondialdehyde; Nrf2, nuclear factor erythroid 2-related factor 2; DCFH-DA, diacetyldichlorofluorescein; MMP, mitochondrial membrane potential; SLC7A11, solute carrier family 7 member 11; GPX4, glutathione peroxidase 4; GSH, glutathione; GSSH, glutathione sulfate; con, control.

To further explore the potential mechanism of GluOC alleviating ferroptosis in TNBC, in vitro experiments with MDA-MB-231 cells transfected with si Nrf2 were performed. Protein expression levels of Nrf2, GPX4, and SLC7A11 were significantly reduced in the si Nrf2 group compared with the blank control group, and GluOC mitigated these effects (Figure 3a,b. The uncropped blots are shown in Figure S1D). MDA and GSH levels were measured after knocking down Nrf2, and it was demonstrated that GluOC prevented the increase in MDA levels caused by knocking down Nrf2 (Figure 3c) and mitigated the decrease in GSH levels caused by si Nrf2 (Figure 3d). Nrf2 knockdown also increased MMP in MDA-MB-231 cells, while GluOC treatment mitigated the effect of si Nrf2 (Figure 3e). Subsequently, it was demonstrated that the addition of GluOC interfered with si Nrf2-induced lipid peroxidation (Figure 3f). Additionally, si Nrf2 increased ROS levels in MDA-MB-231 cells, but the addition of GluOC decreased ROS levels (Figure 3g). This suggested that the sensitivity of MDA-MB-231 cells to ferroptosis increased after Nrf2 knockdown, while GluOC decreased the sensitivity of MDA-MB-231 cells to ferroptosis by promoting the Nrf2/SLC7A11/GPX4 axis.

### 3.4. GluOC Promotes SLC7A11 and GPX4 Expression Through the WWP1/PTENPI3K/AKT/Nrf2 Signaling Pathway

**Figure 4:**
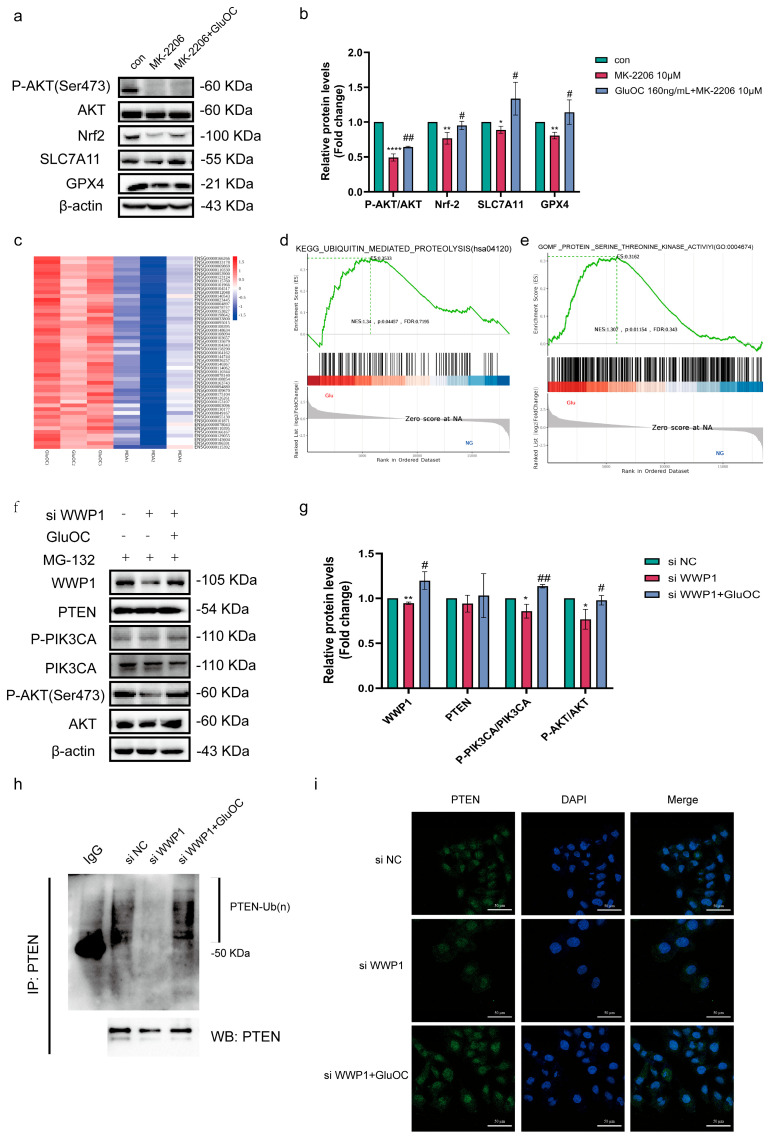
GluOC inhibits MDA-MB-231 breast cancer ferroptosis via the WWP1/PTEN/PIK3CA/AKT signaling pathway. (**a**) GluOC alleviated the expression of P-AKT (Ser 473), Nrf2, SLC7A11, and GPX4 in MDA-MB-231 cells induced by MK-2206. (**b**) Western blotting used ImageJ software for density quantification. Data are presented as the mean ± SD (n = 3). * *p* < 0.05, ** *p* < 0.01, **** *p* < 0.0001 con vs., MK-2206. # *p* < 0.05, ## *p* < 0.01, MK-2206 vs. GluOC+MK--2206. (**c**) Heat map. (**d**) GSEA analysis showed that MDA-MB-231 gene was significantly enriched in UPS pathway after GluOC treatment. (**e**) GSEA analysis showed that MDA-MB-231 gene was significantly enriched in tyrosine kinase activation pathway after GluOC treatment. (**f**) GluOC alleviated the expression of WWP1, P-PIK3CA, and P-AKT (Ser 473) in MDA-MB-231 cells induced by si WWP1. (**g**) Western blotting used ImageJ software for density quantification. (**h**) GluOC alleviated the ubiquitination of PTEN in MDA-MB-231 cells induced by si WWP1. (**i**) Subcelluar localization of PTEN in si NC or si WWP1 and/or GluOC. Confocal images of si NC or si WWP1 and/or GluOC stained with DAPI (blue) and PTEN (green) (scale bar = 50 μm). Data are presented as the mean ± SD (n = 3). * *p* < 0.05, ** *p* < 0.01 con vs., si WWP1. # *p* < 0.05, ## *p* < 0.01, si WWP1 vs. GluOC. GluOC, uncarboxylated osteocalcin; WWP1, WW domain-containing E3 ubiquitin protein ligase 1; PIK3CA, phosphatidylinositol-4,5-bisphosphate 3-kinase catalytic subunit α; P, phosphorylated; Nrf2, nuclear factor erythroid 2-related factor 2; SLC7A11, solute carrier family 7 member 11; GPX4, glutathione peroxidase 4; si, small interfering RNA; GSEA, gene set enrichment analysis; con, control.

To explore the specific mechanism by which GluOC inhibits ferroptosis, the expression levels of several proteins involved in ferroptosis were analyzed. AKT can inhibit ferroptosis by regulating Nrf2 [18]. Subsequently, the activity of Nrf2 was inhibited, and the protein expression levels of Nrf2, SLC7A11 and GPX4 were measured. These results showed that after adding the AKT phosphorylation inhibitor MK-2206, the protein expression levels of Nrf2, SLC7A11, and GPX4 decreased after P-AKT (Ser 473) was decreased (Figure 4a,b. The uncropped blots are shown in Figure S1E). The results of GSEA gene enrichment showed that GluOC promoted the enrichment of ubiquitin–proteasome system (UPS) (Figure 4c,d) and serine/threonine kinase activity (Figure 4e). It has been reported that the E3 ubiquitination ligase WWP1 can ubiquitinate PTEN, inhibit its activity, and activate the PIK3CA/AKT signaling pathway to increase the occurrence and development of cancer [19]. After reducing the expression of WWP1, it was demonstrated that the expression level of PTEN did not change, but the expression levels of P-PIK3CA and P-AKT (Ser 473) were reduced. After GluOC was added, the effect of si WWP1 (Figure S2) was reversed, and the PIK3CA/AKT signaling pathway was activated (Figure 4f,g. The uncropped blots are shown in Figure S1F). The IP experiment further verified GluOC ubiquitination of PTEN via WWP1 (Figure 4h). In addition, laser confocal assays showed that si WWP1 increased the membrane recruitment of PTEN, while GluOC increased the expression levels of PTEN in the nucleus (Figure 4i). GluOC ubiquidized PTEN via WWP1, thereby further activating the PIK3CA/AKT/Nrf2 signaling pathway, increasing the expression of SLC7A11 and GPX4 proteins, and inhibiting ferroptosis in MDA-MB-231 BC cells.

### 3.5. GluOC Increases TCA Circulation Through SLC38A1, Which, in Turn, Promotes Cancer Development

**Figure 5:**
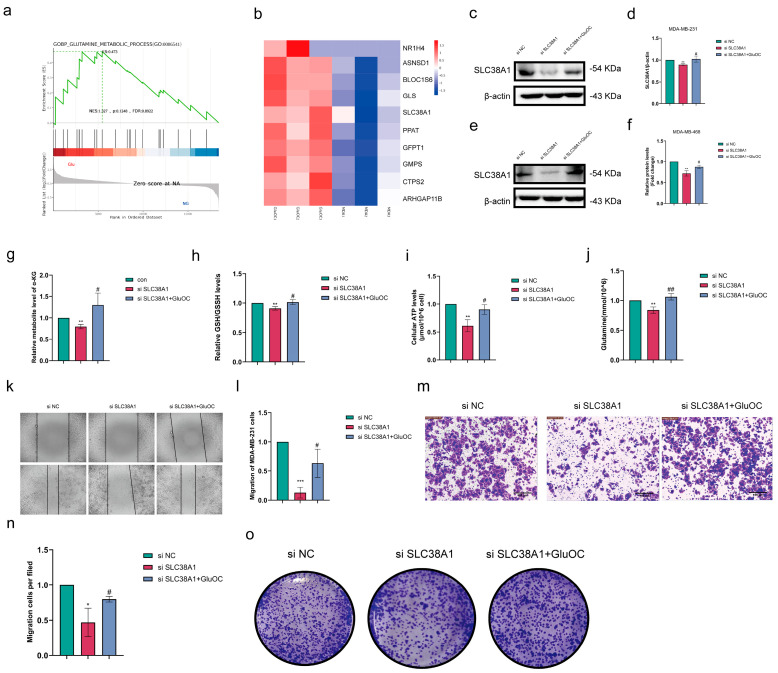
GluOC regulates glutamine metabolism in MDA-MB-231 cells through SLC38A1. (**a**) GSEA analysis showed that MDA-MB-231 gene was significantly enriched in glutamine metabolism pathway after GluOC treatment. (**b**) Heat map of glutamine metabolic pathway. (**c**) GluOC decreased the expression levels of SLC38A1 in MDA-MB-231 cells induced by SLC38A1 knockdown. (**d**) Western blotting of MDA-MB-231 cells used ImageJ software for density quantification. (**e**) GluOC decreased the expression levels of SLC38A1 in MDA-MB-468 cells induced by SLC38A1 knockdown. (**f**) Western blotting of MDA-MB-468 cells used ImageJ software for density quantification. (**g**) Cellular α-KG level. (**h**) Cellular GSH level. (**i**) Cellular ATP level. (**j**) Cellular Gln levels. (**k**) Wound healing assay results showing the migratory capacity of cells (scale bar = 100 μm). (**l**) Quantification of wound healing assay. (**m**) Transwell assay results indicated the cell migration capacity (scale bar = 100 μm). (**n**) Quantification of Transwell assay. (**o**) The clonogenicity of stably transfected MDA-MB-231 cells showed cell proliferation. Data are presented as the mean ± SD (n = 3). * *p* < 0.05, ** *p* < 0.01, *** *p* < 0.001 con vs. si SLC38A1. # *p* < 0.05, ## *p* < 0.01 si SLC38A1 vs. GluOC. GluOC, uncarboxylated osteocalcin; SLC38A1, solute carrier family 38 member 1; GSEA, gene set enrichment analysis; α-KG, α-ketoglutaric acid; GSH, glutathione; Gln, glutamine; con, control; si, small interfering RNA.

DEG and GSEA analysis based on genomic results from MDA-MB-231 cells (con and GluOC groups) showed that the glutamine gene SLC38A1 was differentially expressed in the two differential genomes (Figure 5a,b). To clarify the relationship between GluOC and SLC38A1, the effect of GluOC on glutamine metabolism was investigated. These results showed that GluOC increased the expression levels of SLC38A1 in MDA-MB-231 (Figure 5c,d. The uncropped blots are shown in Figure S1G) and MDA-MB-468 cells (Figure 5e,f. The uncropped blots are shown in Figure S1G. In MDA-MB-231 cells, silencing SLC38A1 decreased the levels of α-KG (Figure 5g), GSH (Figure 5h), ATP (Figure 5i), and glutamine (Figure 5j), whilst GluOC treatment alleviated the effects of si SLC38A1. These results suggested that GluOC regulated glutamine metabolism in TNBC via SLC38A1. Subsequently, the reduction of SLC38A1 reduced the migration capacity of MDA-MB-231 cells. By contrast, the addition of GluOC hindered the loss of cell migration induced by si SLC38A1 (Figure 5k–o). It was further demonstrated that GluOC promoted malignant progression of MDA-MB-231 BC cells through SLC38A1-dependent glutamine metabolism.

### 3.6. GluOC Promotes Tumor Growth and Metastasis and Inhibits Ferroptosis

**Figure 6:**
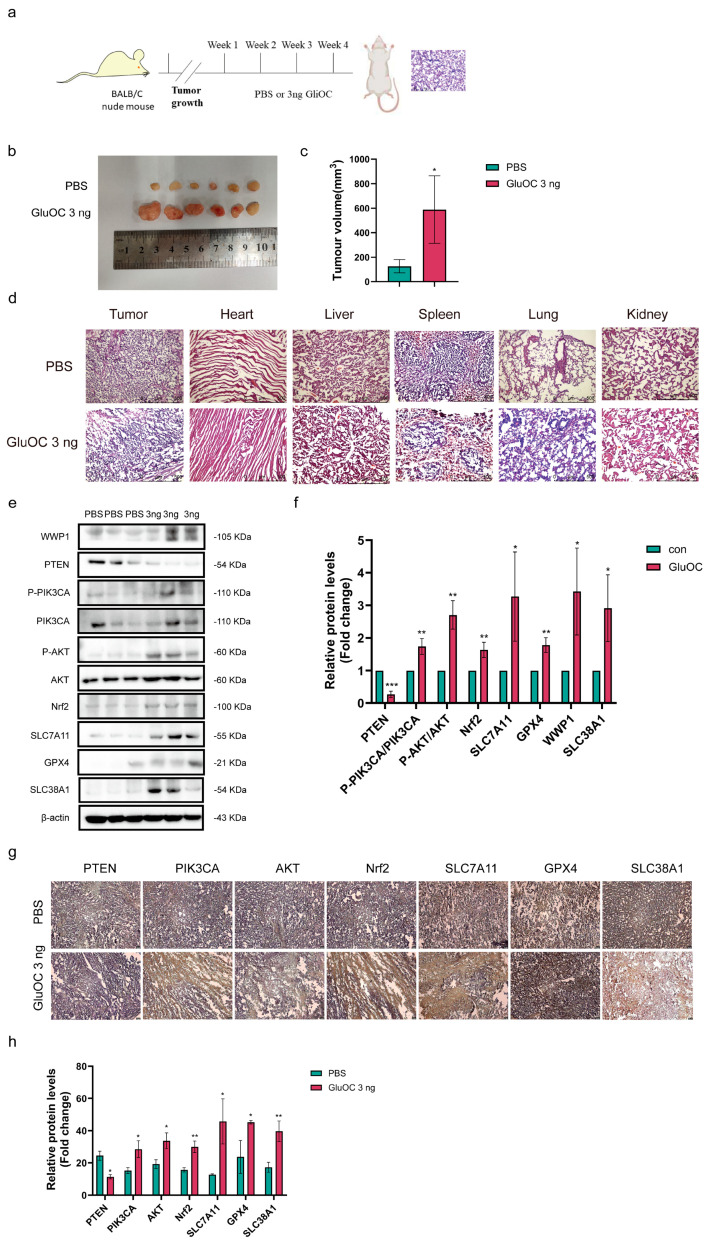
GluOC promotes tumor growth in nude mice. (**a**) Flowchart of the handling process of nude mice. (**b**) Tumors in the nude mice. (**c**) Quantification of tumor volume (n = 4, largest diameter of the tumor is 17 mm, and the largest volume is 1836.77 mm^3^). (**d**) Hematoxylin and eosin staining of the heart, liver, spleen, lung, and kidney of mice. (**e**) GluOC increased the expression levels of WWP1, P-PIK3CA, P-AKT, Nrf2, SLC7A11, GPX4, and SLC38A1 in tumors. (**f**) Western blotting of tumor tissue used ImageJ software for density quantification. (**g**) Protein expression levels in different treatment groups were assessed through immunohistochemistry. (**h**) Quantification of IHC. Data are presented as the mean ± SD (n = 3). * *p* < 0.05, ** *p* < 0.01, *** *p* < 0.001 con vs. 3 ng GluOC. GluOC, uncarboxylated osteocalcin; WWP1, WW domain-containing E3 ubiquitin protein ligase 1; P, phosphorylated; PIK3CA, phosphatidylinositol-4,5-bisphosphate 3-kinase catalytic subunit α; Nrf2, nuclear factor erythroid 2-related factor 2; SLC7A11, solute carrier family 7 member 11; GPX4, glutathione peroxidase 4; SLC38A1, solute carrier family 38 member 1; si, small interfering RNA; con, control.

A tumor bearing model in nude mice was constructed. MDA-MB-231 cells were inoculated into the subcutaneous skin of nude mice, and PBS and 3 ng GluOC were injected adjacent to the tumor daily (Figure 6a). After 20 days, tumors were collected for follow-up experiments. These results showed that 3 ng GluOC significantly promoted tumor growth (Figure 6b,c). In addition, the heart, liver, spleen, lung, and kidney were collected for H&E staining, and these results showed that the lung cell wall thickened after GluOC treatment (Figure 6d). Subsequent Western blotting results showed that the protein expression levels of WWP1, P-PIK3CA, P-AKT, Nrf2, SLC7A11, GPX4, and SLC38A1 in the GluOC-treated group were increased, but the protein expression level of PTEN was decreased (Figure 6e,f. The uncropped blots are shown in Figure S1H,I). In addition, tumor tissues were analyzed using immunohistochemistry, and these results showed that GluOC reduced the expression levels of PTEN but increased the expression levels of P-PIK3CA, P-AKT, Nrf2, SLC7A11, GPX4, and SLC38A1 (Figure 6g,h). These results suggested that GluOC increased the expression of WWP1, thereby inhibiting PTEN and activating the PIK3CA/AKT signaling pathway, increasing the expression levels of SLC7A11 and GPX4 and inhibiting ferroptosis of tumor cells. In addition, GluOC increased the expression levels of the amino acid transporter SLC38A1, which promoted the TCA process by increasing the levels of α-KG, further promoting tumor growth and metastasis and inhibiting ferroptosis of TNBC.

## 4. Discussion

**Figure 7:**
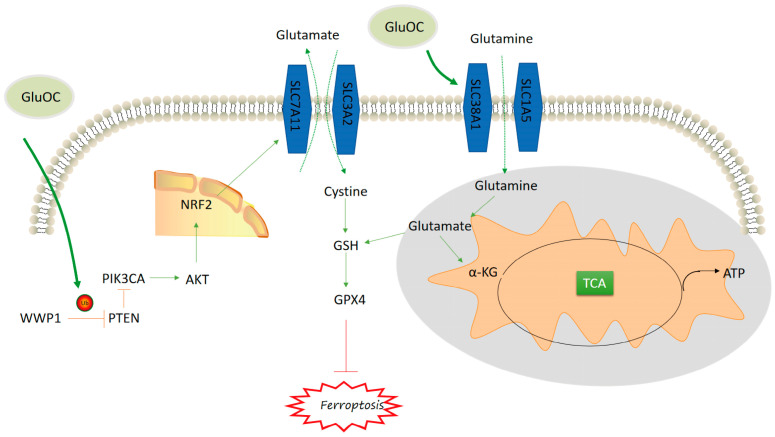
Mechanism diagram of GluOC inhibiting ferroptosis in TNBC.

Dixon et al. [20] first reported ferroptosis as a novel method of cell death. Ferroptosis can affect certain types of cancer such as breast, liver, and ovarian cancer [21]. The present study reported that GluOC promoted TNBC proliferation and metastasis, while inhibiting ferroptosis. GluOC promoted the WWP1/PTEN/PIK3CA/AKT signaling pathway in TNBC, thereby increasing the expression levels of SLC7A11 and GPX4. Additionally, GluOC induced the expression levels of SLC38A1 and increased the TCA flux and ATP content in TNBC cells (Figure 7).

SLC7A11, a member of the SLC family, promotes the synthesis of GSH, whereas inhibition of its expression induces ferroptosis [17,22]. SLC38A1 is a glutamine transporter, which serves a crucial role in the uptake of essential amino acids [23]. It has been reported that SLC7A11 and SLC38A1 expression is upregulated in BC tissues and cells, and high SLC7A11 and SLC38A1 expression levels are associated with a poor prognosis in patients with BC [24]. SLC7A11 mRNA expression levels and cystine consumption are higher in ER- BC cells compared with ER+ BC cells [25]. In the present study, the mRNA profiles of untreated and GluOC-treated MDA-MB-231 cells were compared using RNA sequencing analysis. SLC7A11 and SLC38A1 expression levels in the GluOC-treated group were significantly increased. Subsequently, MDA-MB-231, MCF7, and SKBR3 BC cells were treated with GluOC, and it was demonstrated that the expression levels of SLC7A11 and SLC38A1 in MDA-MB-231 cells were elevated. GPX4 is a downstream effector molecule of SLC7A11 [26]. SLC7A11 and GPX4 affect lipid peroxidation activity by regulating the production of GSH, thereby inhibiting ferroptosis [27]. It was demonstrated that GluOC can alleviate reduced SLC7A11 and GPX4 expression levels induced by si SLC7A11 in MDA-MB-231 and MDA-MB-468 cells by increasing SLC7A11 expression and inhibiting ferrous ion accumulation in cells. GluOC also increased GSH levels via SLC7A11 in MDA-MB-231 cancer cells, thereby preventing ferroptosis of MDA-MB-231. Therefore, GluOC inhibited ferroptosis in TNBC cells.

The expression of SLC7A11 is regulated by several signaling pathways, including the Nrf2 pathway [28,29]. Nrf2 is a major regulator of the activity of several ferroptosis-related and lipid peroxidation-related proteins, such as GSH synthesis and metabolism via SLC7A11 and GPX4 [30]. The results of the present study showed that GluOC increased the expression levels of GPX4 and SLC7A11 through the Nrf2/SLC7A11/GPX4 axis, alleviated the increase in MDA and the decrease in GSH content induced by si Nrf2, and blocked the increase in MMP, lipid peroxidation, and ROS induced by si Nrf2. GluOC stabilized the redox state of MDA-MB-231 cancer cells and inhibited ferroptosis.

Nrf2 is an important regulatory factor in cellular resistance to oxidative stress, and ROS can activate Nrf2 through the PI3K/AKT signaling pathway [31,32], thus regulating ferroptosis [33]. It has been previously reported that cancers with PIK3CA mutations are sensitive to PI3K inhibitors [19,34]. The results of the present study showed that GluOC increased the expression levels of Nrf2 by activating the PIK3CA/AKT signaling pathway, thereby increasing the expression of SLC7A11 and GPX4 and increasing GSH content in MDA-MB-231 BC cells, promoting TNBC proliferation and metastasis whilst inhibiting ferroptosis.

By inhibiting the phosphorylation of PI3K, PTEN inhibits its activity and blocks downstream AKT signaling [35]. UPS is a dynamic system that synergistically regulates protein stability and activity through ubiquitination and deubiquitination [36]. WWP1 activates the PI3K/AKT pathway by inhibiting PTEN activation, which is closely related to TNBC, when loss or inhibition of PTEN expression can be observed [35,37]. It was demonstrated that GluOC increased ubiquitination of PTEN by upregulating WWP1 expression, thereby activating the PI3K/AKT/SLC7A11 axis and reducing the sensitivity of TNBC to ferroptosis.

Glutamine is a nutrient used by tumor cells, and the uptake of glutamine depends on the expression levels of glutamine transporters SLC1A5 or SLC38A1 on the cell membrane [38,39]. It has been previously reported that SLC38A1 may serve an important role in lung, breast, liver, colorectal, and endometrial cancers [40,41,42,43]. Intracellular glutamine is deaminated to produce glutamate under the catalysis of glutaminase, which is the substrate for the synthesis of α-KG, an intermediate of the tricarboxylic acid cycle [44,45]. Glutamate is also involved in the synthesis of glutathione, and GSH serves a role in maintaining intracellular redox homeostasis [46]. GSEA results showed that GluOC promoted glutamine metabolism. The present study demonstrated that GluOC alleviated the decrease in glutamine content, GSH content, and ATP caused by the decrease in SLC38A1. Additionally, GluOC promoted the migration ability of MDA-MB-231 BC cells by increasing SLC38A1 expression.

The in vivo study results showed that compared with the PBS group, GluOC-treated nude mice had increased tumor volume and white nodules in the lungs, indicating that GluOC promoted the growth and lung metastasis of TNBC cells. Western blotting results showed that the expression levels of ferroptosis resistance proteins WWP1, P-PIK3CA, P-AKT, Nrf2, SLC7A11, and GPX4 were increased, but the protein expression levels of PTEN were decreased. GluOC increased the expression levels of SLC7A11 through the WWP1/PTEN/PIK3CA/AKT signaling pathway and also increased the expression levels of SLC38A1, promoted the proliferation and metastasis of TNBC, and inhibited ferroptosis in vivo.

Transcriptomic analysis uncovered that 597 DEGs were upregulated, while 8 were downregulated (polyploid logFoldChange ≥ 2, and *p* < 0.05). KEGG pathway enrichment analysis indicated that pathways such as ferroptosis, apoptosis, cell cycle, fatty acid metabolism, and other metabolic pathways were impacted. Notably, the arachidonic acid signaling pathway was altered during this process. Fatty acid metabolism furnishes a crucial lipid substrate for ferroptosis and modulates the cell’s sensitivity to ferroptosis. In subsequent research, we will delve deeper into whether GluOC can counteract ferroptosis by affecting fatty acid metabolism in TNBC. KEGG pathway analysis revealed that GluOC inhibits the death of TNBC cells and optimizes their metabolic processes via multiple signaling pathways, including the P53 and AMPK signaling pathways and so on. Nevertheless, additional research is essential to clarify whether GluOC regulates the expression of SLC7A11 and other genes through alternative targets. Another aspect that requires further exploration is whether fatty acid metabolism exerts regulatory effects on the metabolism of other amino acids. Moreover, it remains to be investigated whether GluOC plays a role in TNBC immunomodulation and chemotherapy resistance. This study lays a solid foundation for in-depth research into the mechanism underlying GluOC-promoted TNBC proliferation and metastasis.

One limitation of the present study is that although GluOC was found to play an important role in TNBC proliferation and migration through the ROCK1 signaling pathway, the number of lung metastases of TNBC was not counted. They were not counted because of the application of lung tissue to experiments such as HE staining. Pulmonary nodule quantification will be performed in subsequent experiments to more precisely demonstrate that GluOC promotes TNBC lung metastasis. In addition, the small sample size of nude mice used in this study, as well as physiological and genetic differences between mice and humans, are limitations of this study; therefore, further research should consider the potential role of GluOC levels in TNBC patients’ bodies on cancer growth and metastasis in clinical practice.

## 5. Conclusions

In summary, the present study demonstrated that GluOC activated the WWP1/PTEN/PIK3CA/AKT signaling pathway to increase the expression levels of SLC7A11 and SLC38A1, increase GSH levels, and inhibit ferroptosis of TNBC cells, which promoted TNBC proliferation and metastasis. Our work provides insights for achieving treatments based on understanding TNBC’s anti-mortality properties. The results of the present study may provide a valuable theoretical basis for understanding the regulation of ferroptosis in BC cells and be used in the development of novel therapeutic targets in the future.

## Figures and Tables

**Table 1:** siRNA sequence.

Gene	Forward Primer (5′ to 3′)	Reverse Primer (5′ to 3′)
si NC	UUCUCCGAACGUGUCACGUTT	UUCUCCGAACGUGUCACGUTT
si SLC7A11-1498	GCUGAUUUAUCUUCGAUACAATT	UUGUAUCGAAGAUAAAUCAGCTT
si SLC7A11-901	CCCUGGAGUUAUGCAGCUAAUTT	AUUAGCUGCAUAACUCCAGGGTT
si SLC7A11-544	CCUGUCACUAUUUGGAGCUUUTT	AAAGCUCCAAAUAGUGACAGGTT
si Nrf-2	CGCUCAGUUACAACUAGAUTT	AUCUAGUUGUAACUGAGCGTT
si WWP1	GAAGTCATCTGTAACTAAA	GCAGAGAAATACTGTTTAT
si SLC38A1	CAGUGACAUUGCUGUCUAUAUTT	AUAUAGACAGCAAUGUCACUGTT

**Table 2:** Primers for reverse transcription-quantitative PCR.

Gene	Forward Primer (5′ to 3′)	Reverse Primer (5′ to 3′)
Solute carrier family 7 member 11	TGCTGGGCTGATTTATCTTCG	GAAAGGGCAACCATGAAGAGG
WWP1	ACAGTGGCAATCTCAGCG	GCAAAGGTCCATAAGGGT
β-actin	AGAGAGGCATCCTCACCCTG	GATAGCACAGCCTGGATAGCA

## Data Availability

The data generated in the present study may be found at the following URL: https://dataview.ncbi.nlm.nih.gov/object/PRJNA1197700?reviewer=hhgrg2t0f5kj94e45jhgnhpfln (accessed on 1 January 2025).

## Supplementary Materials

The following supporting information can be downloaded at: https://www.mdpi.com/article/10.3390/cancers17050739/s1, Figure S1A Western blot full size image of MDA-MB-231 and MCF7 cells. The red box indicates the area of grouping and calculation. Figure S1B Western blot full size image of MDA-MB-231 cells were grouped into si NC, si SLC7A11 and si SLC7A11+GluOC. Figure S1C Western blot full size image of MDA-MB-468 cells were grouped into si NC, si SLC7A11 and si SLC7A11+GluOC. Figure S1D Western blot full size image of MDA-MB-231 cells. It is divided into three components: si NC, si Nrf2 and si Nrf2+GluOC. Figure S1E Western blot full size image of MDA-MB-231 cells. It is divided into three components: con, MK-2206 and MK-2206+GluOC. Figure S1F Western blot full size image of MDA-MB-231 cells. It is divided into three components: si NC, si WWP1 and si WWP1+GluOC. Figure S1G Full-size Western blot image of MDA-MB-231 and MDA-MB-468 cells. It is divided into three components: si NC, si SLC38A1 and si SLC38A1+GluOC. Figure S1H and I Full-size images of western blots of tumor. As shown in Figure S1H and I, tumor-bearing mice were divided into PBS and 3ng GluOC, showing the full-size western blot images of animal tumor tissue. Figure S2: The gene levels of WWP1 in MDA-MB-231 cells before and after siRNA injection were determined by qRT-PCR.

## Abbreviations

The following abbreviations are used in this manuscript:

BC	breast cancer
TNBC	triple-negative BC
PR	progesterone receptor
ER	estrogen receptor
HER2	epidermal growth factor receptor 2
OC	osteocalcin
GluOC	uncarboxylated osteocalcin
DEGs	differentially expressed genes
OS	overall survival
GSEA	gene set enrichment analysis
MDA	malondialdehyde
GSH	glutathione
UPS	ubiquitin–proteasome system
TCA	tricarboxylic acid
PIP3	phosphatidylinositol triphosphate
