# Supplementary material for: GluOC Induced SLC7A11 and SLC38A1 to Activate Redox Processes and Resist Ferroptosis in TNBC
Source: Cancers (Basel). 2025 Feb 21;17(5):739. doi: 10.3390/cancers17050739 (PMC11899354; doi:10.3390/cancers17050739)
Supplement: Supplementary file 1 [file cancers-17-00739-s001.zip › cancers-3460792-supplementary.pdf]

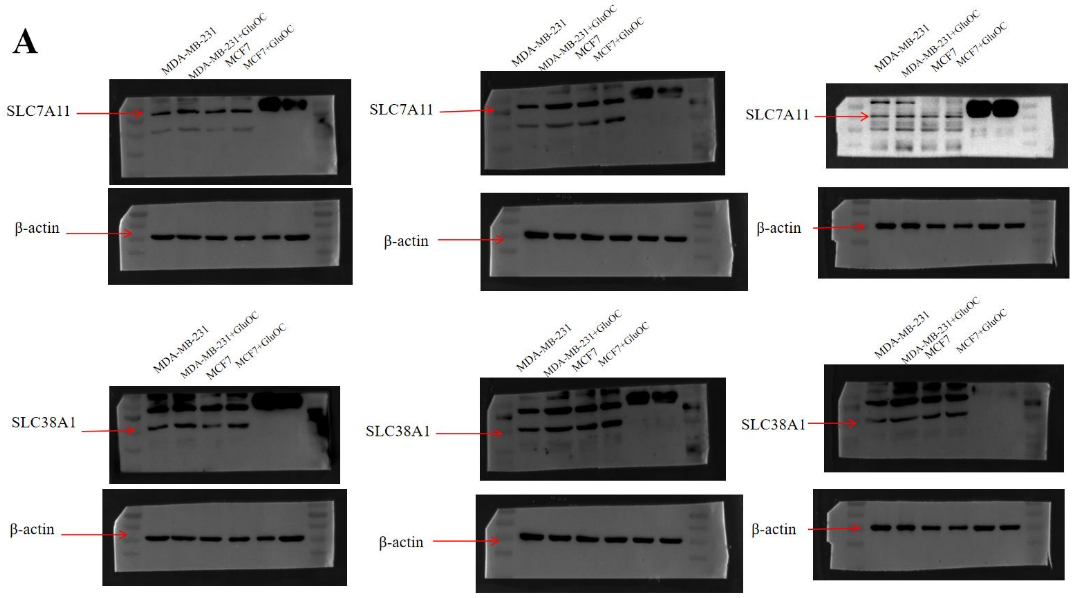

Figure S1A Western blot full size image of MDA-MB-231 and MCF7 cells. Figure shows the cancer cells were grouped into con and 160 ng/mL GluOC. The full-size western blot images of SLC7A11 and SLC38A1 were used to evaluate GluOC's effect in cancer cells.

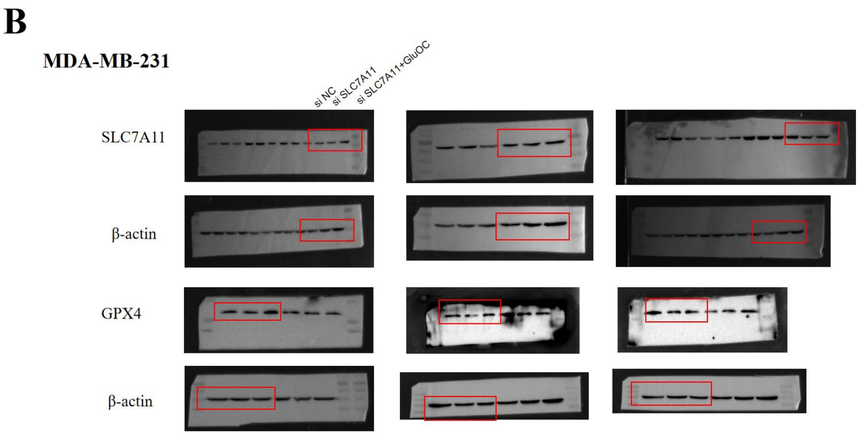

Figure S1B Western blot full size image of MDA-MB-231 cells were grouped into si NC, si SLC7A11 and si SLC7A11+GluOC. The full-size western blot images of SLC7A11 and GPX4 were used to evaluate GluOC's effect in TNBC cells. The red box indicates the area of grouping and calculation.

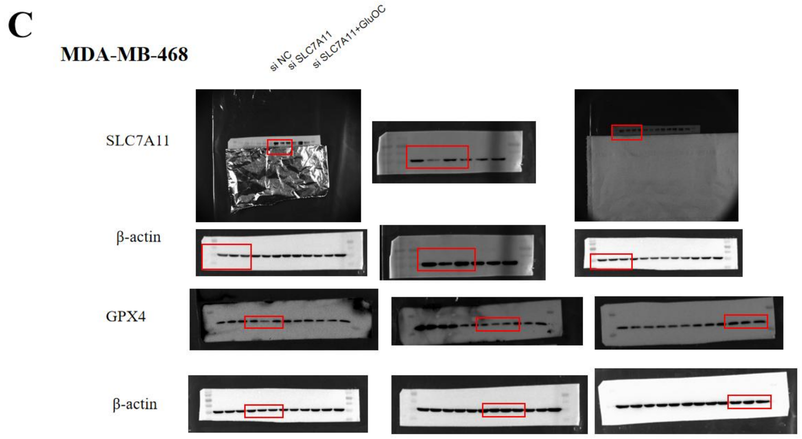

Figure S1C Western blot full size image of MDA-MB-231 and MDA-MB-468 cells. Figure shows the MDA-MB-468 cells were grouped into si NC, si SLC7A11 and si SLC7A11+GluOC. The full-size western blot images of SLC7A11 and GPX4 were used to evaluate GluOC's effect in TNBC cells. The red box indicates the area of grouping and calculation.

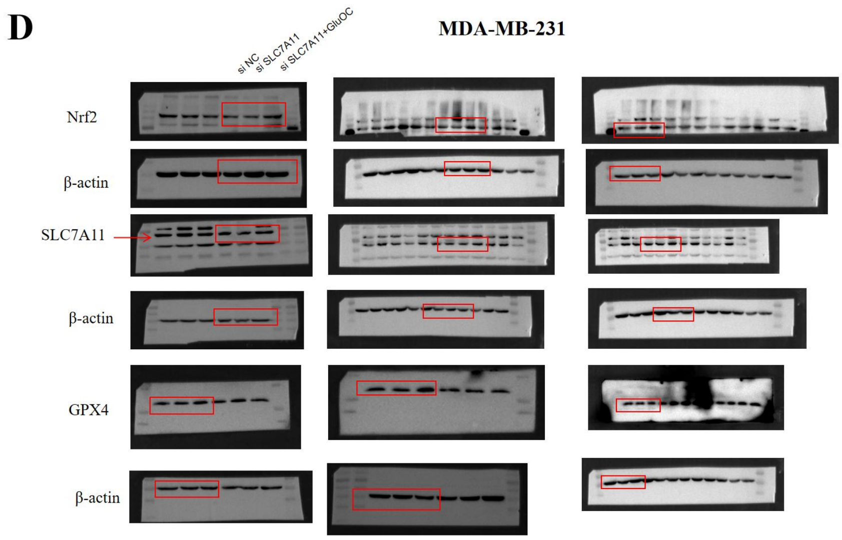

Figure S1D Western blot full size image of MDA-MB-231 cells. Figure is Nrf2, SLC7A11 and full-size western of GPX4 in MDA-MB-231 cells. It is divided into three components: si NC, si Nrf2 and si Nrf2+GluOC. The red box of a full-size western blot image used for grouping and calculation.

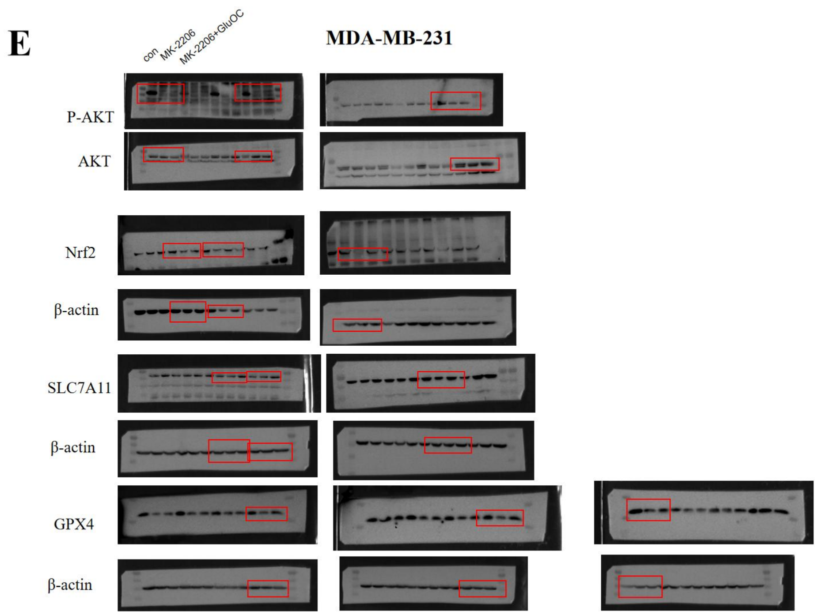

Figure S1E Western blot full size image of MDA-MB-231 cells. Figure is P-AKT, Nrf2, SLC7A11 and full-size western of GPX4 in MDA-MB-231 cells. It is divided into three components: con, MK-2206 and MK-2206+GluOC. The red box of a full-size western blot image is the area for grouping calculations.

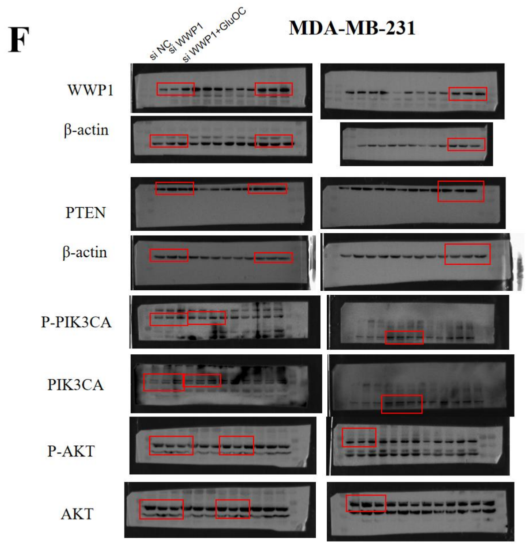

Figure S1F Western blot full size image of MDA-MB-231 cells. Figure shows full-size western blot images of WWP1, PTEN, P-PIK3CA, PIK3CA, P-AKT and AKT in MDA-MB-231 cells. It is divided into three components: si NC, si WWP1 and si WWP1+GluOC. The red box of a full-size western blot image is the area for grouping calculations.

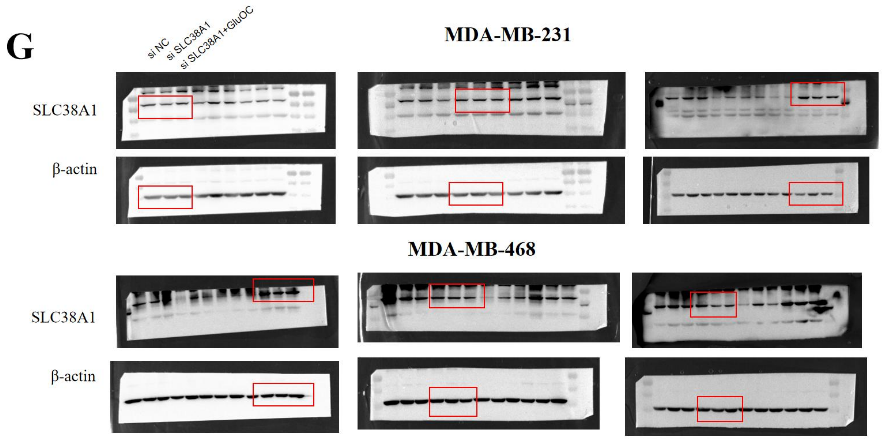

Figure S1G Full-size Western blot image of MDA-MB-231 and MDA-MB-468 cells. Figure shows full-size western blot images of SLC38A1 in MDA-MB-231 and MDA-MB-468 cells. It is divided into three components: si NC, si SLC38A1 and si SLC38A1+GluOC. The red box of a full-size western blot image is the area for grouping calculations.

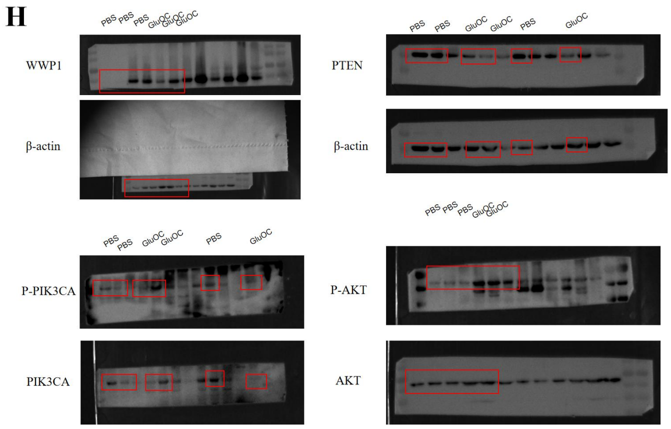

Figure S1H Full-size images of western blots of tumor. As shown in figure **H**, tumor-bearing mice were divided into three components, PBS and 3ng GluOC, showing the full-size western blot images of animal tumor tissue. Here is the expression level of WWP1, PTEN, P-PIK3CA and P-AKT proteins in tumor tissue. The red boxes indicate the cropped regions.

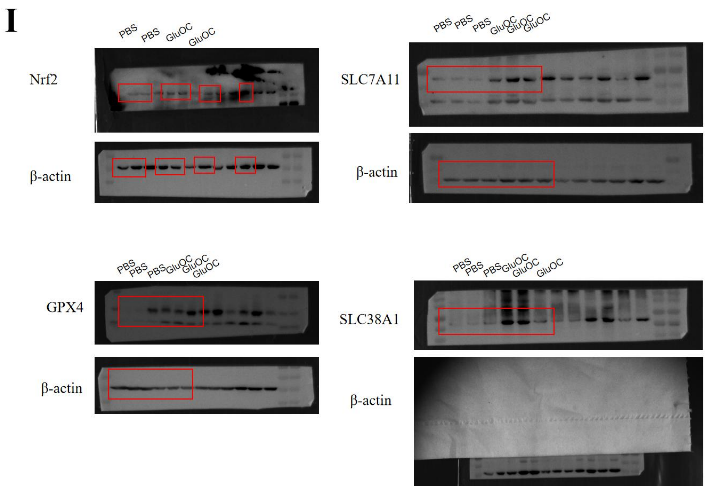

Figure S1I Full-size images of western blots of tumor. As shown in figure **I**, tumor-bearing mice were divided into three components, PBS and 3ng GluOC, showing the full-size western blot images of animal tumor tissue. Here is the expression level of Nrf2, SLC7A11, GPX4 and SLC38A1 proteins in tumor tissue. The red boxes indicate the cropped regions.

In the process of electrical transfer of proteins, multiple proteins were transfected from different parts of the same SDS-PAGE gel using pre-stained protein marker as reference. In this process, we used membrane regeneration solution to incubate different antibodies in PVDF membrane again. We have provided three independent repeats and submitted the whole western blot picture as an attachment. In addition, we labeled the molecular weight of the protein in the figures.

Figure S2

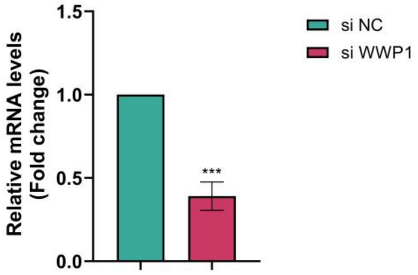

Figure S2 The gene levels of WWP1 in MDA-MB-231 cells before and after siRNA injection were determined by qRT-PCR. The results represent at least three

independent experiments, and the data are presented as the mean±SD (n=3). ***p < 0.001 compared with the si NC group.

Supplementary method

Total RNA was extracted from the MDA-MB-231 breast cancer using TRIzol® Reagent according the manufacturer’s instructions (Magen). Paired-end libraries were prepared using a ABclonal mRNA-seq Lib Prep Kit (ABclonal, China) following the manufacturer's instructions. Sequencing was performed with an Illumina Novasea 6000 /MGISEO-T7 instrument. The data generated from Illumina/BGl platform were used for bioinformatics analysis. Then clean reads were separately aligned to reference genome with orientation mode using HISAT2 software (http://dachwankimlab.github.io/hisat2) to obtain mapped reads. Feature Counts (http://subread.sourceforge.net/) was used to count the reads numbers mapped to each gene. And then FPKM of each gene was calculated based on the length of the gene and reads count mapped to this gene. Differential expression analysis was performed using the DESeq2 (http://bioconductor.org/packages/release/bioc/html/DESeq2.htm), Differential expression genes (DEGs) with $|\log_2 FC| > 1$ and P-adj < 0.05 were considered to be significantly different expressed genes. The Gene Ontology (GO) and Kyoto Encyclopedia of Genes and Genomes (KEGG) enrichment analysis of differential genes can explain the functional enrichment of differential genes and clarify the differences between samples at the gene function level. ClusterProfiler R software package was used to GO function enrichment and KEGG pathway enrichment analysis. When p-value < 0.05, it is considered that the GO or KEGG function is significantly enriched.
